# The Clinical Society

**Published:** 1897-10-16

**Authors:** 


### THE CLINICAL SOCIETY.

At the opening meeting of the Clinical Society held last Friday, a case of Loretta's operation for stricture of the pylorus was reported by Dr. W. H. White and Mr. Langton. The patient was a man aged 32, who had for some years suffered from gastric pain, which was increased by food and relieved by vomiting. There had been much loss of flesh. Nothing definite could be detected on examination. He had had typhoid fever when 18 years of age. After Loretta's operation quick recovery had taken place, and he had gained flesh rapidly. The operation had taken place four years ago, and the patient now seemed in perfect health. The stenosis of the pylorus was probably inflammatory. In the discussion it was pointed out that Loretta's operation was not popular in this country, and that pyloroplasty was the better operation.

A case of œsophagotomy for the removal of an impacted tooth-plate, in which cellular emphysema developed in the tissues of the neck without injury to the pleura, was related by Mr. Makins. It was thought that by the opening of the tissues of the mediastinum a certain in-sucking of air from without had taken place into these tissues, and that during expiration this was driven into the cellular tissue of the neck.

Dr. Phear related a case of cerebral sinus thrombosis in a child. The obvious symptoms began suddenly, without any evidence of ear disease or history of recent specific fever.

